# Inference of Gene Regulatory Networks Using Time-Series Data: A Survey

**DOI:** 10.2174/138920209789177610

**Published:** 2009-09

**Authors:** Chao Sima, Jianping Hua, Sungwon Jung

**Affiliations:** Computational Biology Division, Translational Genomics Research Institute, Phoenix, AZ 85004, USA

## Abstract

The advent of high-throughput technology like microarrays has provided the platform for studying how different cellular components work together, thus created an enormous interest in mathematically modeling biological network, particularly gene regulatory network (GRN). Of particular interest is the modeling and inference on time-series data, which capture a more thorough picture of the system than non-temporal data do. We have given an extensive review of methodologies that have been used on time-series data. In realizing that validation is an impartible part of the inference paradigm, we have also presented a discussion on the principles and challenges in performance evaluation of different methods. This survey gives a panoramic view on these topics, with anticipation that the readers will be inspired to improve and/or expand GRN inference and validation tool repository.

## 1. INTRODUCTION

Biological system has been traditionally studied *via *reductionism approach, that is, explaining cell behaviors by studying functions of individual cellular components. Though the knowledge being insightful, it has been increasingly apparent that the understanding of the complex cellular system requires understanding of how different components work together. The advent of high-throughput technology like microarrays, where cellular activities can be measured at genome-wide scale, has provided just this platform and thus created an enormous interest in mathematically modeling biological network, particularly gene regulatory network (GRN). The goal is to mimic the biological network in some abstract level, and a better understanding of the underlying biological system could be achieved through the analysis on the resulted mathematical model. To do so, it is critical to have a reliable modeling and inference procedure.

Amid the explosion of efforts in inferring biological network models that has been witnessed by the last decade, there have been accordingly a number of reviews on these different methodologies. Some of these reviews are focused on one specific type of modeling, for example, on Bayesian Network and Dynamic Bayesian Network (graphical model) [1, 2], or topologies (random *v.s.* scale-free *v.s.* hierarchical) [3]. Others describe models in different categories: Cho *et al*. reviewed methods that incorporate prior knowledge as well as machine learning approaches (e.g., Genetic Algorithm or Neural Net) [4], in addition to the general methods we reviewed in this paper; Schlitt *et al*. ordered the modeling methods according to their complexity [5], from simple ones that are just an aggregation of different functional components, to those that are trying to model the dynamics and have a myriad of parameters to tune. van Someren *et al*. took a unique approach, listing the models in a chronological order along with pointing out the key differences between them [6]. Lastly there is a good review by de Jong [7] which provided a concise mathematical background and formulation for many widely used models.

A lot of those early efforts have been focused on non-temporal data, largely due to the paucity of time-series data. As the biological system is inherently complex and GRN is essentially an ensemble of genes which evolve over time, time-series data will clearly capture a more thorough picture of the system than non-temporal data do, which only take *snapshots* of the system at one given time point. Table **1** lists some of the time-series data sets that have been used. (Bar-Joseph has a general review of gene expression time-series data [26]). It is therefore our interest to present a survey of study on models and inference methodologies for time-series data, its promises and unique challenges. In addition to our focus of time-series data inference, there are two other distinctive aspects of our review work that are different from others: (1) we will take a practitioner's view, reviewing different implementations within one modeling framework as well as different modeling frameworks; (2) we have a special interest in performance evaluation: the validation of the methodologies and comparison studies where it is possible. It is our hope that at the end of paper, readers will have a rather complete knowledge of the methods in the literature, and be inspired to improve and/or expand this tool repository.

This paper is organized as follows: modeling and inference methods focusing on inferring the structure of the network will be reviewed in Section 2, which include the *Relevance Network*, *Bayesian Network* and *Dynamic Bayesian Network* (DBN). This is followed by Section 3 in which methods inferring both structure and dynamics will be reviewed. Among them, *Boolean Network* and *Probabilistic Boolean Network* (PBN) have their states in discrete space and *Markov Model* (MM), *State Space Model* (SSM), and *Ordinary Differential Equation* (ODE) typically have their states in continuous space. The prominent characteristics of these models are shown in Fig. (**1**). After a discussion on performance evaluation of the models in Section 4, we conclude the paper in Section 5.

We need to point out that the placement of DBN in Section 2 is due to its close kinship with *Bayesian Network* and we feel it is more natural to introduce them together. It would have well fallen into Section 3 otherwise. In fact DBN was shown to be related to a lot of models in Section 3: Lähdesmäki *et al*. showed that variables in both discrete-valued DBN and PBN can have the same joint probability distribution [27]; DBN can also be considered as a generalization of Boolean Network [28], or SSM [29].

## 2. INFERRING STRUCTURE OF THE NETWORK

Given a set of genes as nodes for a network, the structure is basically the assemble of all the interconnections among the nodes. Depending on the models, these connections usually take on different meanings, but generally specify the relationships between one gene with another gene, or another set of genes. *Relevance Network*, argued by some to be *ad hoc* network, is the simplest model in this category.

### 2.1. Relevance Network

Relevance networks can be categorized as networks in which relationship among genes can be defined using a pairwise measure of relevance. Given a set of *n* genes **G** = {*g*_1_,*g*_2_,...,*g_n_*} and a set of observations **D** on genomic profiling (e.g., gene expression) for *m* time points, a relevance between *g_i_* and *g_j_* can be evaluated using their time-series profiles, [*g*_*i*,1_ *g*_*i*,2_ ... *g_i,m_*] and [*g*_*j*,1_ *g*_*j*,2_ ... *g_j,m_*]. Various relevance measures have been used to infer relationships between two genes, from simple correlation measures to biologically motivated relevance measures.

One example of using a simple Pearson correlation measure is the work of Remondini *et al*., where they used two sets of gene expression data from rat fibroblast cell lines to construct correlation-based networks [30, 31]. Even though such a correlation measure can be useful in many cases, it cannot provide causal information. To overcome this limitation, Gupta *et al*. used a slope metric (SR) to elucidate not only the presence of a relationship between two genes but also its directionality [32], which is used to represent causality. In their study, the structure was determined using a correlation measure with a preferred threshold. The directionality of relevance was determined using the following SR, based on the assumption that a gene *g_i_* is linearly dependent on another gene *g_j_*, i.e., *g_i_* = *a_ij_* + *b_ij_g_j_*.

(1) $$\mathrm{SR}=\frac{\min\left(\left|b_{\mathrm{ij}}\right|,\left|b_{\mathrm{ji}}\right|\right)}{\max\left(\left|b_{\mathrm{ij}}\right|,\left|b_{\mathrm{ji}}\right|\right)}$$

If $\mathrm{SR}=\left|b_{\mathrm{ij}}\right|/\left|b_{\mathrm{ji}}\right|$, then the directionality is determined as *g_i_* → *g_j_* and if $\mathrm{SR}=\left|b_{\mathrm{ji}}\right|/\left|b_{\mathrm{ij}}\right|$, then it becomes *g_j_* → *g_i_*. This interpretation of the directed edge between two genes is based on the biological assumption that a small change in the source gene is associated with a large change in the target gene. This method was applied to a time-series microarray data of *B. subtilis* [21] and showed some correspondence with already known biological information.

Besides the approaches based on relevance measures for the same time point, there are approaches to incorporate time-delay in measuring the relevance between genes. Schmitt *et al*. used a time-lagged correlation to infer gene regulatory networks [19]. For a transcription profile represented by a series of *m* measurements taken at equally spaced time points, the correlation between genes *g_i_* and *g_j_* with a time lag, ***τ***, is **R**(***τ***) = *r_ij_*(***τ***), defined by

(2) $$S_{\mathrm{ij}}=\sum_{t=\tau +1}^{m}\left(g_{i,t}-\bar{g_{i}}\right)\left(g_{j,t-\tau }-\bar{g_{j}}\right)$$

(3) $$r_{\mathrm{ij}}\left(\tau \right)=\frac{S_{\mathrm{ij}}\left(\tau \right)}{\sqrt{S_{\mathrm{ii}}\left(\tau \right)S_{\mathrm{jj}}\left(\tau \right)}}$$

where *g_i,t_* denotes the expression of a gene *g_i_* at time *t*, $\bar{g_{i}}$ is the averaged expression value of a gene *g_i_* across all time points, and *S_ij_* is essentially the inner product between the time-shifted profiles. This measure was used to analyze whole genome DNA microarrays of *Synechocystis* under various light intensity conditions [19]. Seed genes were selected first based on the time-lagged correlation between the expression profile and the light intensity profile across the time points. These seed genes were expanded using the time-lagged correlation between the average expression profile of seeds and the expression profile of each gene.

Ma *et al*. and Barker *et al*. used a biologically motivated approach to measure the dependency between two genes using temporal information in time-series profiles [33-35]. After ternarizing the expression data into highly/averagely/lowly expressed states (denoted by H/A/L respectively), Ma *et al*. looked for dependencies between *g_i_* being ‘H’ at one time and *g_j_* also being ‘H’ at next time point [33, 34]. Specifically, they computed a statistics on the difference between the number of occurrences of *g_i_* being ‘H’ at one time AND *g_j_* also being ‘H’ at next time point, and the expected number of these occurrences. The latter was computed by averaging the product of the number of *g_i_* being ‘H’ at one time (regardless of *g_j_* 's state at next time point) and number of *g_j_* being ‘H’ at next time point (regardless of *g_i_*'s state at previous time point). A 95 percent confidence level was then used to determine whether the dependency is deemed significant. Using this method, the authors constructed a gene interaction diagram from a yeast data set [10]. Similarly, Barker *et al*. proposed a method to determine whether *g_i_* is regulated by *g_j_*, based on three ratios of samples, the ratio of *g_j_* activating *g_i_*, the ratio of *g_j_* repressing *g_i_* and the ratio of *g_j_* doing nothing on *g_i_* [35]. With threshold values to those three ratios, the final structure of a GRN is constructed. These methods investigate the relationship between two time-series profiles with single epoch-delay, thus assuming Markov condition regarding the time-point, where an observation at some time point is dependent on that of the previous time-point only.

Kwon *et al*. proposed a method based on string alignment to infer transcriptional regulation relationships from time-series gene expression data, in which relevance with arbitrary time-delay can be considered [36]. They converted the time-course of each gene into a string composed of rising(R), constant(C) or falling(F). The similarity between two event strings is evaluated by string alignment algorithm using scoring matrix that describes similarity score between each pair of event characters. This method was applied to a yeast cell-cycle data [10] and showed it could find some already known transcriptional regulation relationships.

There were also efforts to enhance the quality of inferred relevance network through post processing using additional criteria. Bickel used decisive false discovery rate (dFDR) to estimate the probability of spurious connections between genes in GRNs [37]. After building a network using a time-lagged correlation measure, three kinds of probabilities were evaluated for each edge, i) the probability of being a false positive connection, ii) the probability of being a connection with wrong time order and iii) the probability of being a connection with a time-delay while there is actually no time-delay. This method was applied to yeast cell-cycle data [10] and showed it could successfully find already known genetic relationships.

### 2.2. Bayesian Network

A Bayesian network model is a graphical representation of a joint probability distribution of random variables. A Bayesian network *B* is defined as a tuple of (*G*,**Φ**), where *G* is a graph structure that represents conditional dependency relationships between random variables and **Φ** is a set of parameters describing conditional probability distribution. In modeling GRNs, *G* corresponds to the topology of a GRN, where each node represents a gene as a random variable and each edge represents dependency between genes. With Markov assumption on dependency relationships, the joint probability distribution of genes **G** = {*g*_1_,*g*_2_,...,*g_n_*} is described as follows:

(4) $$P\left(g_{1},g_{2},...,g_{n}\right)=\Pi_{i=1}^{n}P\left(g_{i}\left|\mathrm{Pa}\right.\left(g_{i}\right),\theta \right)$$

where **Pa**(*g_i_*) is a set of parents of *g_i_* in *G* and *θ* is a statistics from **D** . In this framework, a Bayesian network represents a static joint probability distribution of a set of random variables. For this reason, most of applications of Bayesian networks do not incorporate the temporal information in time-series data. Usual assumption in using Bayesian networks for time-series data is that the time-series is from the stationary state of the target biological system. One may use the temporal information to determine the direction of edges in Bayesian networks. However, it is important to notice that the direction of an edge in Bayesian networks does not necessarily represent causality between random variables.

Learning a Bayesian network *B* = (*G*,**Φ**) from observed data **D** implies learning the dependency structure *G* and learning the set of probabilistic parameters **Φ**. Learning **Φ** is a relatively easy problem once *G* and **D** are given, and learning *G* can be done by finding G^*^ with maximum *P*(*G* | **D**) However, learning *G* given **D** is a hard problem because the number of possible graph structures increase exponentially as the size of a network increases. For this reason, most of approaches using Bayesian networks focus on small problems or take heuristics to handle large problems. One popular heuristic approach is restricting the *G* to a certain category.

One of the first applications of Bayesian networks for genetic networks is the work by Friedman *et al*. with the strategy of restricting *G* [38]. In this study, learning Bayesian networks was applied to microarray gene expression data for cell-cycle of *S. cerevisiae* [10]. By using Bayesian network learning, the authors analyzed gene expression data for Markov relation between genes and the coverage of influence for each gene, where the coverage of influence was measured by counting the number of descendants in the graph structure for that gene. They used sparse candidate algorithm, which restricts the candidate parents of each node in *G* during the search. In their study, 800 genes related to cell-cycle were considered as random variables. Their result was evaluated through literature mining and statistical significance test. Even though they used time-series expression data, temporal information was not used in their study due to the previously mentioned reason.

Considering the problem of learning Bayesian networks as an optimization problem for the objective function *P*(*G* | **D**), search algorithms for large solution spaces can be also used. One of such methods is an estimation of distribution algorithm (EDA) and Dai *et al*. used the EDA for learning genetic networks with a Bayesian network model [39]. With EDA, they evolved a population of *G* s that have high Bayesian information criterion (BIC) scores. After some iterated evolution process, *k* graph structures with highest scores were chosen to build final aggregated genetic networks. Their method was applied to two sets of time-series data [10, 11], and was evaluated by Gene Ontology (GO) search and literature mining.

### 2.3. Dynamic Bayesian Network

A Dynamic Bayesian Network (DBN) is an extension of a Bayesian network model to incorporate temporal concept. Compared to conventional Bayesian networks, DBNs include random variables $\left\{g_{1,t-1},g_{2,t-1},...,g_{n,t-1}\right\}$ of time step *t*–1 in addition to $\left\{g_{1,t},g_{2,t},...,g_{n,t}\right\}$ of time step *t* . A transition network is composed of those 2*n* random variables with no edges from time step *t* to *t*–1. It is assumed that the transition probability $P\left({\vec{g}}_{t}|{\vec{g}}_{t-1}\right)$, where ${\vec{g}}_{t}$ represents the values of *n* genes at time *t* , is homogeneous across entire observation. Learning DBNs can be done using the same idea of learning Bayesian networks. The only difference is that we need to consider additional random variables of time *t* – 1. From this perspective, Friedman *et al*. extended some scoring rules for learning structures from Bayesian networks to the case of DBNs [40].

The difficulty in learning DBNs is its heavy computational complexity. Because additional *n* random variables are considered compared to conventional Bayesian networks, learning algorithms should consider much more candidate graph structures and probabilistic parameters. For this reason, most of applications of DBN usually target smaller systems compared to the study of Bayesian networks. Further, heuristics to restrict candidate graph structures are widely used in the applications of DBNs.

Ong *et al*. used DBNs to infer regulatory network for tryptophan metabolism in *E. coli* [41]. By using prior knowledge (operon map), they restricted possible network structures into predetermined category. Then a DBN was learned using Expectation-Maximization (EM) method with gene expression data of 8 time points [16]. Missal *et al*. used mutual information between two gene expression profiles and applied *χ*^2^-test for the significance of the mutual information to determine the structure of a DBN [42]. Zhao *et al*. took a similar approach of using mutual information [43], but they used minimum description length (MDL) principle [44] to determine the threshold of the significance, and to remove indirect or false links in a post-pruning process. However, by using different encoding schemes, this method can generate non-unique results that need *ad hoc* adjustment. Dougherty *et al*. overcome this drawback by measuring the description length based on a universal model: normalized maximum likelihood model [45]. Zou *et al*. used DBNs with various time-delay, by shifting time-series profiles with properly predicted amount of time steps [46], and applied their method to the yeast cell-cycle data of Chou *et al*. [11].

Variables in DBNs can take continuous values as well as discrete values. When random variables are continuous in a DBN, conditional probability tables, which are used in the discrete case, cannot be used. To model *P*(*g_i,t_* | **Pa**(*g_i,t_*)) in a continuous domain, Kim *et al*. assumed a nonparametric additive regression model with Gaussian noise [47-49],

(5) $$g_{i,t}=m_{i1}p_{i1,t-1}+...+m_{\mathrm{iq}}p_{\mathrm{iq},t-1}+\varepsilon_{i}\left(t\right)$$

where *q* is the number of parents of *g_i,t_*, *p_ij_* is the *j* th parent of *g_i,t_* and *ε_i_*(*t*) is a Gaussian noise of *g_i_* at time *t* . A scoring measure for DBN structures was proposed based on the regression model. This method was applied to *yeast* cell-cycle data of Spellman *et al*. [10]. A nonlinear regression extension was proposed by Ferrazzi *et al*. in which *p_ij,t–1_* in Eqn. (5) is replaced by *tanh*(*p_ij,t–1_*), where *tanh*(.) is the hyperbolic tangent function [50].

Several recent studies focused on using different types of gene expression data. Dojer *et al*. proposed a method to handle perturbed gene expression data in using DBNs [51]. In their approach, candidate regulators for each gene were inferred from only a subset of entire data, where the target gene was not perturbed. The motivation of this approach is based on the assumption that data with a specific gene perturbed may not be used in the process of inferring regulators of that gene, because knocking out a gene can disable regulations *toward* that gene. But since knocking out a gene may represent under-expression of that gene, the targets of a perturbed gene may still have regulation effect *from* the perturbed gene. The effectiveness of this method was shown using simulated data from an ordinary differential equation model. Lähdesmäki *et al*. proposed a method for learning DBNs from mixture of steady state data and time-series data [52]. If a steady state data is **D***_A_* and a time-series data is **D***_π_*, learning a DBN structure *G* requires the evaluation of the marginal likelihood *P*(**D***_A_*,**D***_π_* | *G*). By assuming **D***_A_* and **D***_π_* are independent given (*G*,**Φ**), where **Φ** is a set of probabilistic parameters, the marginal likelihood can be evaluated as follows:

(6) $$P\left(D_{A},D_{\pi }\left|G\right.\right)=\int_{\Phi }P\left(D_{A},D_{\pi }\left|G\right.,\Phi \right)P\left(\Phi \left|G\right.\right)d\Phi$$

(7) $$=\int_{\Phi }P\left(D_{A}\left|G,\Phi \right.\right)P\left(D_{\pi }\left|G,\Phi \right.\right)P\left(\Phi \left|G\right.\right)d\Phi$$

Evaluation of *P*(**D***_A_* | *G*,**Φ**) can be done in the same way of static Bayesian networks and the evaluation of *P*(**D***_π_* | *G*,**Φ**) is done using the steady-state distribution of the DBN.

## 3. INFERRING STRUCTURE AND DYNAMICS

Structure alone does not completely describe the network. Often we are interested in the evolution of the system from a given condition, or the response to a particular perturbation, which require a network model that is able to characterize the dynamics and describe the system transitions into future time. The states, defined as the values for the vector of genes, can be either in continuous domain or constrained to be in discrete space.

### 3.1. Discrete State Space

A discrete state space model characterizes a system using quantized data. The most popular approaches are Boolean network and probabilistic Boolean network (PBN).

Boolean network assumes that the gene expression takes just two levels: ON/1 and OFF/0, and the functional relationship between the genes is determined by logical rules. A Boolean network consists of *n* nodes **G** = {*g*_1_,*g*_2_,...,*g_n_*} and a list of Boolean functions **F** = {*f*_1_,*f*_2_,...,*f_n_*}. Each node $g_{i}\in \left\{0,1\right\}$ is a binary variable that represents the state (expression) of gene *i* . The Boolean function *g_i,t+1_* = *f_i_*(**Pa**(*g_i,t_*)) specifies how the value of node *g_i_* at next time point *t* + 1 is determined by the values of its input nodes **Pa**(*g_i_*) at current time point *t* .

REVEAL [53] proposed by Liang *et al*. is one of the first Boolean-network-based inference scheme. Based on information theory, if the mutual information of the input and output is equal to the entropy of the output, the input fully determines the output. Hence for each node, REVEAL searches for the minimal input node set that can fully determine the output. Rather than using mutual information, Akutsu *et al*. chose to check consistency for all logic rules [54], and later extended the inference to noisy data [55]. Furthermore, Akutsu *et al*. proved that, if the maximum number of input nodes is bounded by a constant and transition pairs are randomly picked, the sample size needed to reconstruct the original Boolean network is in *O*(log *n*) [54]. However, for limited sample size, there exist considerable number of valid networks that are consistent with the given data. Rather than pick one or a few networks, Martin *et al*. enumerate all the consistent networks and aggregate them by network's attractors [24]. Their results show that most networks share only a few common attractors, which indicate similar network dynamics.

Some view the binary-state system like Boolean network as oversimplification that has significant information loss. Laubenbacher and Stigler introduced a multi-state system where the gene-gene relationship is determined by the computational algebra of the finite field [56]. Normally the solution is not unique, and the inference scheme will pick the minimal network functions by removing all redundant terms. Like REVEAL, this approach assumes noiseless data and the performance suffers when noise presents.

The Boolean network and the multi-state extension are based on the assumption of deterministic gene-gene relationship. Probabilistic Boolean network adds a sense of randomness into the Boolean network by allowing the nodes to have more than one associated Boolean functions. So for a PBN, the nodes **G** 's associated functions are now denoted as **F** = {**F**_1_,**F**_2_,...,**F***_n_*}, where ${\text{F}}_{i}=\left\{f_{1}^{\left(i\right)},f_{2}^{\left(i\right)},...,{f_{l}}_{\left(i\right)}^{\left(i\right)}\right\}$, i.e., each node's output is now associated with *l_(i)_* possible Boolean functions. At any time point, PBN allows each node *i* to take only one Boolean function from **F***_i_*. Hence there are altogether $\Pi_{i=1}^{n}l_{\left(i\right)}$ realizations of a PBN. Perturbation probability and selection probability were later introduced to allow the network be perturbed in current realization or switched between realizations, respectively.

It was suggested in Shmulevich *et al*. that *Coefficient of Determination* can be used to infer the network [57]. Later Marshall *et al*. implemented an inference procedure for PBN that successfully infers all the constituent Boolean networks, at well as all the perturbation and selection probabilities associated with them [58]. Unfortunately, as they pointed out in the paper, the amount of temporal data needed for inference is huge. A more practical way to infer PBN is to approximate the network by multivariate Markov model, as shown by Ching *et al*. [59]. In this model, the state of gene *i* at time point *t* takes a binary probability distribution denoted by vector ${{\vec{p}}_{i,t}=\left[P\left(g_{i,t}=0\right),P\left(g_{i,t}=1\right)\right]}^{\prime }$. The model assumes

(8) $${\vec{p}}_{i,t+1}=\sum_{j=1}^{n}\lambda_{\mathrm{ij}}T_{\mathrm{ij}}{\vec{p}}_{j,t},$$

where *T_ij_* is the probability transition matrix from gene *j* to gene *i* , and *λ_ij_* the non-negative weight factor that has $\sum_{j=1}^{n}\lambda_{\mathrm{ij}}=1$.

### 3.2. Continuous State Space

A continuous state space model can characterize a system without discretizing the data, a step argued to result loss of information. One of the straightforward modeling strategies here is to describe the system evolution as a *Markov Process*, and this gives the *Markov Model* networks.

#### 3.2.1. Markov Model

Dewey *et al*. studied a simple linear Markov model in the form of

(9) $${\vec{g}}_{t+1}=T{\vec{g}}_{t}$$

in which ${\vec{g}}_{t}$ denotes the gene expression levels at time *t* , and the matrix *T* is the transition for genes between two time points [60, 61]. Assuming a total of *m* time points, this model can be written in another form

(10) $$G_{+1}=\mathrm{TG}$$

where $G_{+1}=\left[{\vec{g}}_{2}{\vec{g}}_{3}...{\vec{g}}_{m}\right]$ and $G=\left[{\vec{g}}_{1}{\vec{g}}_{2}...{\vec{g}}_{m-1}\right],$ and the transition matrix *T* can be calculated as *G*_+1_*G*^+^, *G*^+^ being the pseudo-inverse of *G* obtained by using *singular value decomposition* (SVD). They further extended it to include non-linear terms that capture both between-time (contained in *GG^T^* ) and between-gene (contained in *G^T^G* ) correlations by considering the form *G*_+1_ = *T*_1_*G* + *T*_2_*G^T^G* + *GG^T^T*_3_, as well as higher order Markov terms [60].

Holter *et al*. performed SVD [62-64] on the data $D=\left[{\vec{g}}_{1}{\vec{g}}_{2}...{\vec{g}}_{m}\right]=U\sum V^{T}$ and took the first *r* rows in **Σ***V^T^*, *r* being the number of non-zero eigenvalues of *DD^T^*, as the *dominant patterns*, or *modes* [65]. The temporal expression for each gene is therefore a linear combination of these *modes* ${\vec{X}}_{i}=\left[x_{i,1}x_{i,2}...x_{i,m}\right],i=1,...,r.$. The linear Markov model they considered is in the form of

(11) $$\left(\begin{array}{c} x_{1,t+1}\\ \underset{\begin{array}{c} \cdot \\ \cdot \\ \cdot \end{array}}{x_{2,t+1}}\\ x_{r,t+1} \end{array}\right)=T\left(\begin{array}{c} x_{1,t}\\ \underset{\begin{array}{c} \cdot \\ \cdot \\ \cdot \end{array}}{x_{2,t}}\\ x_{r,t} \end{array}\right)$$

The *modes* transition matrix *T* is then estimated by minimizing the divergence of the trajectory from the observed values.

Wiggins *et al*. took a Bayesian approach and rewrote the transition dynamics with added term of Gaussian noise ${\vec{\varepsilon }}_{t}$ as [66]:

(12) $${\vec{g}}_{t+1}-{\vec{g}}_{t}=\left(T-I\right){\vec{g}}_{t}+{\vec{\varepsilon }}_{t}$$

where *I* is the identity matrix. Given the biological prior which was modeled in *T* , they were able to derive the posterior probability for *T* and used the expectation of *T* to represent the transition dynamics. They also considered an augmented model which treated latent variables as additional “hidden degrees of freedom” and derived the expected *T* after integrating out these latent variable.

Another variation of Markov model was studied by Li *et al*. in which the transition of gene expression *g_i,t_* is modeled as power functions:

(13) $$g_{i,t+1}=\alpha_{i}\underset{j}{\Pi }g_{j,t}^{w_{\mathrm{ij}}}+\left(1-\beta_{i}\right)g_{i,t}+\varepsilon_{i,t}$$

where *α_i_* is the rate of transcription and *β_i_* is the rate of degradation [67, 68]. To find the most-likely structure of the network, the authors used either guided simulated annealing method [67, 69] or Genetic Algorithm [68] to optimize a score function which is essentially the likelihood function with a penalty term that penalizes complex models (complex in the sense that number of parameters being too large). Used on a set of yeast *Saccharomyces Cerevisiae* microarray data [10], this procedure recovered 31 out of 42 and 17 out of 22 regulation relationships which are consistent with those found experimentally [67, 68].

#### 3.2.2. State Space Models

State Space Model (SSM) can be viewed as an extension to Markov models, based on the assumption that gene expression levels are hidden states and cannot be directly observed, and are related with the observed values by some transformation. A general linear *SSM with input* takes the form as:

(14) $${\vec{g}}_{t+1}=T{\vec{g}}_{t}+A{\vec{u}}_{t}+{\vec{\varepsilon }}_{g,t}$$

(15) $${\vec{y}}_{t}=C{\vec{g}}_{t}+B{\vec{v}}_{t}+{\vec{\varepsilon }}_{y,t}$$

where $\vec{g}$ denotes the state of the genes for the system, $\vec{y}$ the observed data for $\vec{g}$, *T* the state transition matrix, *C* the state to observation matrix, and *A* and *B* are the inputs influence matrices for inputs ${\vec{u}}_{t}$ and ${\vec{v}}_{t}$, respectively. Here ${\vec{\varepsilon }}_{g,t}$ and ${\vec{\varepsilon }}_{y,t}$ are white noise terms. If *A* = 0 and *B* = 0 , this is reduced to the *basic linear SSM*, or *standard SSM*.

Rangel *et al*. and Beal *et al*. used *SSM with input* and set the inputs as the observations from previous time point [18, 70]. Specifically, their system is described as:

(16) $${\vec{g}}_{t+1}=T{\vec{g}}_{t}+A{\vec{y}}_{t}+{\vec{\varepsilon }}_{g,t}$$

(17) $${\vec{y}}_{t}=C{\vec{g}}_{t}+B{\vec{y}}_{t-1}+{\vec{\varepsilon }}_{y,t}$$

Notice the above equation can be rewritten as

(18) $${\vec{y}}_{t}=C\left(T{\vec{g}}_{t-1}+A{\vec{y}}_{t-1}+{\vec{\varepsilon }}_{g,t-1}\right)+B{\vec{y}}_{t-1}+{\vec{\varepsilon }}_{y,t}$$

(19) $$=\left(\mathrm{CA}+B\right){\vec{y}}_{t-1}+C\left(T{\vec{g}}_{t-1}+C{\vec{\varepsilon }}_{g,t-1}\right)+{\vec{\varepsilon }}_{y,t}$$

Therefore the matrix *T*′ = *CA* + *B* captures the transition in the *observation* domain over time, through the hidden states ${\vec{g}}_{t}$, and the authors focused their interests in *T*′. Rangel *et al*. estimated the model parameters using EM algorithm and constructed confidence interval on *T*′ by using bootstrap, while Beal *et al*. used what they called *Variational Bayesian EM Algorithm*, which can be considered as a Bayesian extension of the standard EM algorithm, to derive a posterior estimation on *T*′.

Both Hirose *et al*. and Yoshida *et al*. argued that the dimension *k* of the hidden states ${\vec{g}}_{t}$, which they called “modules”, is less than the number of dimension for observations ${\vec{y}}_{t}$ [71, 72] . Using *standard SSM* and assuming the noise term ${\vec{\varepsilon }}_{y,t}$ has a diagonal covariance matrix *R* and some other constraints, the authors carefully designed a projection transformation matrix *H* such that the denoised observation vector ${\vec{y}}_{t}^{⋄}=R^{-1/2}\left({\vec{y}}_{t}-{\vec{\varepsilon }}_{y,t}\right)$ can be projected to lower dimension $k\left({\vec{g}}_{t}=H{\vec{y}}_{t}^{⋄}\text{to be exact}\right)$ and the system follows a dynamic as described in:

(20) $${\vec{y}}_{t}^{⋄}=T_{M}{\vec{y}}_{t}^{⋄}+{{\vec{\varepsilon }}_{g^{⋄}}}_{,t}$$

where *T_M_* and ${{\vec{\varepsilon }}_{g^{⋄}}}_{,t}$ are appropriately transformed from *T* and ${\vec{\varepsilon }}_{g,t}$, respectively. All module-module interactions are presented in the transition matrix *T_M_*. Yoshida *et al*. further argued that the network structure may not be the same over time, and they proposed what they called “Markov switching” [71]. In essence, this is an inhomogeneous SSM, where at each time point the system is allowed to change its structure. They put the inference in a Bayesian framework, and introduced an additional vector of hidden variables which served as indicators of whether the system is switching at each time points. Posterior distribution of model parameters were estimated by using Gibbs sampling. Hirose *et al*. on the other hand, did a comparison study with the models in the work by Rangel *et al*. and Beal *et al*. [18, 70] and investigated the benefits of using multiple replicates of time course data.

Kasabov *et al*. also used the *standard SSM* and proceeded to estimate *T* using Kalman filter, and constructed the network from two sets of human leukemic cell line data, which have 32 pre-selected genes and 4 time points [73]. They also showed the potential application in larger data sets with more genes by implementing Genetic Algorithm (fitness function evaluated by using Kalman Filter estimated likelihood) for gene selection. The validation of the networks is rather weak, however, as they merely showed that the observed data fall on the estimated trajectories for four of the genes they selected for the network.

#### 3.2.3. Ordinary Differential Equations

Ordinary differential equations can be used to describe the gene products, e.g., mRNA and protein, and their interactions. A general form of an *n* -node ODE system can be written as:

(21) $$\frac{d\vec{g}\left(t\right)}{\mathrm{dt}}=f\left(\vec{g}\left(t\right),t\right)+S\left(t\right),$$

where $\vec{g}\left(t\right)$ is the gene product concentrations of *n* nodes, $f\left(\vec{g}\left(t\right),t\right)$ the regulation function, and *S(t )* the external stimulus. For genetic regulation network, the most popular ODE model is the linear time-invariant model:

(22) $$\frac{d\vec{g}\left(t\right)}{\mathrm{dt}}=R\vec{g}\left(t\right)+W\vec{s}\left(t\right),$$

where *R* is an *n* × *n* matrix denoting the direct regulation among *n* nodes, and $\vec{s}$ a *k* × 1 constant vector whose effects on *n* nodes are intermediated by the *n* × *k* constant matrix *W* . In practice the available data are sampled at limited time points, so most methods actually solve *Difference Equations* like

(23) $${\vec{g}}_{t+1}-{\vec{g}}_{t}=R{\vec{g}}_{t}+W{\vec{s}}_{t}.$$

In reality, noise, whether significant or slight, is always present in the observed raw data. Some approaches explicitly incorporate the noise into the system as error terms ${\vec{\varepsilon }}_{t}$:

(24) $${\vec{g}}_{t+1}-{\vec{g}}_{t}=R{\vec{g}}_{t}+W{\vec{s}}_{t}+{\vec{\varepsilon }}_{t},$$

Note that this form closely resembles Eqn. (12) in Markov model.

For any node *i* , its regulation is defined by the *i* -th row of *R* , i.e., $R_{i}=\left[r_{\mathrm{i1}},...,r_{\mathrm{in}}\right]$. The signs of $\left[r_{\mathrm{i1}},...,r_{\mathrm{in}}\right]$ determine the network structure: for node *j* , *r_ij_* ≠ 0 means it is an input of node *i* , and depending on whether *r_ij_* is positive or negative, node *j* activates or inhibits node *i* , respectively. If *r_ii_* ≠ 0, then the node *i* is self-regulated. The actual values of [*r_i1_*,...,*r_in_*] determine the production/degradation rates of node *i* . For most algorithms, the objective is to estimate *R* , especially the network structure. Furthermore, they normally decouple the problem and work on the regulation of one gene at a time based on ${\mathrm{dg}}_{i}\left(t\right)/\mathrm{dt}={\;R}_{i}\vec{g}\left(t\right)+W_{i}\vec{s}\left(t\right)$.

Taking $\vec{s}\equiv 0$, Chen *et al*. provided two estimation solutions of *R* [74]. One solution used Fourier transform. It assumed that several cell cycles are observed, and the system is stable enough to be approximated by $\vec{g}\left(t\right)=Qe^{t\lambda }$, where *Q* is constant matrix and *λ* the eigenvalues of *R* . Assuming that each node's input set size is small and fixed, the other solution used Minimal Weight Solutions to Linear Equations [75] to solve the difference equations.

Instead of fixing input set size, de Hoon *et al*. allowed the network to have input sets of different sizes [76]. They assumed that the error terms are normally distributed so log-likelihood of proposed network can be calculated. The search for the network of maximal log-likelihood was regulated by Akaike's Information Criterion (AIC): AIC = -2[log-likelihood] + 2[number of parameters]. The approach limited the size of nodes it can handle to be smaller than the number of time points. Bourque and Sandkoff preferred a forward search approach [77]. For gene *i* , the fitness of its input set is measured by the sum of squared errors (SSE). To add a new input to the existing input set, the decrease of SSE must pass F-test at given significance level. The authors also extended the inference to multiple related networks, where the fitness includes not only SSE, but also evolution cost, which is the sum of pairwise symmetric differences of all networks. Chan *et al*. put connection constraints on linear model through sparse Bayesian learning [78]. In this approach, SSE is regulated by a parameter magnitude function, in which the magnitude of each connection *r_ij_* is weighted by a hyperparameter. The optimization is conducted in a recursive way where the regulation matrix *R* and hyperparameters are estimated alternately.

The linear assumption limits the range of networks the model can emulate. To add nonlinearity to the model, Perkins *et al*. introduced a hybrid model by coupling the concentration value of each node with its logical state [79]. The approach normalizes the concentration to a range of [0, 1], discretizes the production rate to either on or off, and fixes the degradation rate. By this means, the regulation function *f_i_* of gene *i* is reduced to a logical function plus the degradation term.

Another popular non-linear differential equation model is the S-system form, where the production/degradation rate is a product of power-law functions:

(25) $$dg_{i}\left(t\right)/\mathrm{dt}=\alpha_{i}\overset{n}{\underset{j=1}{\Pi }}X_{j}^{u_{\mathrm{ij}}}-\beta_{i}\overset{n}{\underset{j=1}{\Pi }}X_{j}^{v_{\mathrm{ij}}},$$

where *α_i_* and *β_i_* are rate constants, *u_ij_* and *v_ij_* are kinetic orders. The earlier work of Akutsu *et al*. was based on qualitative modeling and used linear programming which can only determine log*α_i_* - log*β_i_* and *u_ij_* - *v_ij_* [55]. Later work like Marino and Voit used Levenberg-Marquardt method to find the parameters that minimize SSE [80]. Daisuke and Horton used distributed genetic algorithm to overcome the local minima [81]. The algorithm is seeded with networks that follow the scale-free property and results in multiple candidates that are later aggregated to determine the network structure. Novikov and Barillot converted differential equations into integral equations :

(26) $$g_{i}\left(t\right)=\sum_{i=1}^{n}\int_{t_{0}}^{t}r_{\mathrm{ij}}\left(t,x\right)g_{j}\left(x\right)\mathrm{dx}+s_{i}\left(t,t_{0}\right),$$

where the *r_ij_* and *s_i_* are modeled by nonlinear time-variant kernel functions: polynomial, exponential or delta-function models [82]. Similar to Bourque and Sandkoff [77], the network is inferred by using forward search, but with *χ^2^* criterion value replacing F-test's p-value.

Recurrent Neural Network (RNN) is another popular model that can capture the nonlinear dynamics of various systems. Xu *et al*. used the model of the following form:

(27) $${\mathrm{dg}}_{i}\left(t\right)/\mathrm{dt}=\left(\sigma \left(R_{i}\vec{g}\left(t\right)+W_{i}\vec{s}+b_{i}\right)-\lambda_{i}g_{i}\left(t\right)\right)/\tau_{i},$$

where *σ*(.) is the sigmoid function, *b_i_* the bias term, *λ_i_* the self-degradation rate, and *τ_i_* the time constant [83]. The network is learned with particle swarm optimization [84]. Busch *et al*. studied the gene regulation network of the migration of human skin keratinocytes with RNN [25]. The model used in their work follows the form

(28) $${\mathrm{dg}}_{i}\left(t\right)/\mathrm{dt}=\left(\sum_{j=1}^{n}r_{\mathrm{ij}}\sigma \left(g_{j}\left(t-\Delta \tau_{j}\right)+b_{i}\right)-g_{i}\left(t\right)+s_{i}\left(t\right)\right)/\tau_{i},$$

where Δ*τ_j_* is the time delay associated with gene *j* . The network is learned with Genetic Algorithm.

Cavelier and Anastassiou pursued a hybrid of linear and nonlinear functions, where linear functions are used for translation and nonlinear functions for transcription [85]. The authors considered three nonlinear functions of increasing complexity: sigmoid-type function, Hill function and thermodynamically derived function. The authors further assumed that the network structure is available through prior knowledge in the literature, so the algorithm focuses on the estimation of the production/degradation parameters through the so-called evolution strategies. Another way to add nonlinearity is through time-delay, as described by Kim *et al*. [86]:

(29) $${\mathrm{dg}}_{i}\left(t\right)/\mathrm{dt}=\sum_{i=1}^{n}r_{\mathrm{ij}}\left(g_{j}\left(t-\tau_{j}\right)+\varepsilon_{\mathrm{ij}}\right),$$

where *ε_ij_* is a noise term.

Rather than inferring the exact chemical kinetic equations regulating every node, Sontag *et al*. estimated the influence of node *i* on the node *j* by measuring the change of node *i* production rate relative to the change of node *j* concentration at any give network status [87]. To do so, they proposed an experiment protocol in which a series of perturbations is applied and the unperturbed and perturbed time-series data are compared and evaluated: for an *n* -gene network, the protocol needs about *n*^2^ perturbations with perfect measurement to fully infer all the influences. Bansal *et al*. also proposed an inference scheme based on perturbation [22]. The difference equation is now written as

(30) $$G_{+1}=\left[R+I,W\right]\left[\begin{array}{c} G\\ S \end{array}\right],$$

where *I* is the identity matrix, $G=\left[{\vec{g}}_{1},{\vec{g}}_{2},...\right]$ , $G_{+1}=\left[{\vec{g}}_{2},{\vec{g}}_{3},...\right]$, and $S=\left[{\vec{s}}_{1},{\vec{s}}_{2},...\right]$ The network structure is then estimated through the dominated singular values of the PCA decomposition of $\left[\begin{array}{c} G\\ S \end{array}\right]$. With PCA decomposition, this approach can handle a lot more genes with limited observations.

Arguing that the accuracy of single best regulation parameter set is prone to error due to limited data, Nam *et al*. aggregated the most likely regulator sets through voting [88]. Similarly, Kim *et al*. used noise injection to improve inference robustness [89]. In their model, *r_ij_* is a time-varying function *r_ij_*(t) = *α_ij_* sin (*ωt* + *φ_ij_*) + *β_ij_*, where *α_ij_*, *ω*, *Φ_ij_* and *β_ij_* are unknown parameters. The effects of gene *j* on gene *i* are assessed on the 2D trajectory map of {*g_i_*(*t*),*r_ij_*(*t*)*g_j_*(*t*)}. By injecting random noise to the original data to generate several slightly different data sets, only the connections that shows certain degree of stability across all data sets are picked.

## 4. PERFORMANCE EVALUATION

With the abundance of proposed models and inference algorithms, it is essential to have a validation protocol so that the merit of each proposed method can be assessed, and guideline can be established in aiding practitioners to choose the right modeling and inference procedure. Validation therefore should be considered as an impartible part of the complete inference scheme.

### 4.1. Distance Measure

Ideally one would compare the inferred network *N_I_*, with ‘ground truth’ network *N_G_*, the one from which data used for inference are derived. The validation is given quantitively in the form of the distance measure *D*(*N_I_*,*N_G_*), as shown in Fig. (**2a**). Two critical issues arise from here, as discussed below.

#### 4.1.1. Lack of ‘Ground Truth’

Although *N_G_* as the underlying biological network is the ultimate golden standard that the inferred network should be evaluated against, unfortunately it is almost guaranteed that we will not be blessed with this knowledge for any biological system of non-trivial size (as a matter of fact, we lack the knowledge for even trivial-sized system, for example, the three gene oscillating network of *E. Coli* [90]). To circumvent this problem, two common approaches have been used.

The first approach is to use ‘partial ground truth’, which is most often in the form of regulation relationships gleaned from literature. This is demonstrated in Fig. (**2b**), in which $D\left(N_{I},N_{G}^{P}\right)$ can be thought as an approximation of *D*(*N_I_*,*N_G_*). This approach has being widely utilized. For example: Hirose *et al*. found genes in each “module” are related to the same molecular function according to Gene Ontology (GO) [72]; in addition to GO, Dai *et al*. also searched *Saccharomyces Genome Database* to find relationships which are consistent to their findings; both Kim *et al*. and Novikov *et al*. compared their yeast cell cycle pathway networks with those selected from KEGG (Kyoto Encyclopedia of Genes and Genomes) [47, 82], and Kim *et al*. further compared with the metabolic pathway reported by DeRisi *et al*. [9]. Although versatile and bearing immediate biological interpretation, this approach is limited by the thoroughness and accuracy of reports from literature, and subject to the bias in mining the literature.

The second approach is the use of synthetic network generator, so we know every aspect of the underlying network $N_{G}^{S}$. Illustrated in Fig. (**2c**), the synthetic network $N_{G}^{S}$ serves as a surrogate of the original *N_G_*, and ideally, should mimic the real biological system. In reality, $N_{G}^{S}$ can only model a subset of the properties in *N_G_* and this leads to a inherent bias where certain class or classes of inference methods could be favored unintentionally. Setting up the synthetic network that is free of this bias is not trivial, but sometimes this obstacle can be overcome by carefully aligning synthetic network properties with those in the study objectives.

One immediate advantage of using $N_{G}^{S}$ is that we can easily study the properties of the proposed inference algorithms. As an example, robustness property can be analyzed by perturbing and adding noise to the network. These learned properties often lead to improvement on the inference. For example, Bansal *et al*. randomly generated 100 networks for each of the two sizes, one with 10 gene and 5 time points, and the other with 1000 gene and 10 time points, and added white Gaussian noise of different standard deviations to the generated time series data [22]. The tuning of parameters in inferring network from real data was facilitated by examining the performances on these synthetic network data. Perkins *et al*. fixed input set size for each node for their ODE model, and randomly selected the elements in input node sets and the associated Boolean functions [79]. They were able to derive the number of data points needed to fully infer the network structure and regulation functions in different scenarios. An intensive simulation study on DBN by Yu *et al*. evaluated various combinations of Bayesian scoring metrics, search heuristics, discretization levels, sampling interval, quantity of data and data interpolation using simulated data from a stochastic linear Markov model [91]. As a result, they published a series of guidelines for various factors in DBN learning. The benefits associated with using synthetic network in validating inference algorithms make it a practical and fruitful choice as surrogate of *N_G_*.

#### 4.1.2. Choice of Criterion

When comparing two networks *N_I_* and *N_G_*, *D*(*N_I_*,*N_G_*) can take one of many possible forms. For example, the most commonly used measures $D\left(N_{I},N_{G}^{P}\right)$ for structures are *true positive* (TP), *true negative* (TN), *false positive* (FP), *false negative* (FN), or some derivation from them (e.g., *Receiver Operating Characteristic*, or *ROC* curves). Dougherty has proposed a list of “semi-metrics” as validation measures for goodness of the inference algorithms [92]. The list can be grown to accommodate different objectives of studies. It is important to notice that there doesn't exist a one-size-fit-all criterion that works as a universal validation measure due to two prominent reasons: (1) the goal and focus vary widely from study to study, and the criterion has to be chosen to be consistent with the objective of the study. For example, if the purpose of the experiment is to discover pairwise gene-gene interactions, then a proper measure could be to compare the difference in connections (either directed or undirected) in *N_I_* and *N_G_*. Or if the interest is in how system evolves, a trajectory-based measure may be more appropriate [92]. (2) Inference algorithms are typically designed for a certain subtype of models, which in turn are proposed for some specific aims of the study. Using a validation measure which is more in line with the same goal will inevitably bias favorably towards these methods. This makes comparing across models particularly difficult, and is part of the reason that we see very few comparative studies on inference methodology. Even when such studies are carried out, they are practically limited to one type of models [93].

### 4.2. Comparative Studies

Some efforts have been made to address the above issues. Brun *et al*. proposed a steady-state trajectory based metric between networks that is independent of nature of networks [94], hence has immediate application in comparative studies. Trajectory of each gene of the network is decomposed into a transient part and a steady-state part, the latter of which could be either constant or periodic, and assessed with an *amplitude cumulative distribution* [95]. *D*(*N_I_*,*N_G_*) is therefore the average (across all genes) of distances, which are computed as some norm between *amplitude cumulative distributions*. This metric could be useful if it is the steady-state behavior of the network that is of interest.

For comparison studies, Hartemink suggested that DBN seems to work better than Bayesian Network [2]. Note the author conceded that this is hardly a conclusion due to the “different properties” of the data used for inference. A more detailed study by Werhli *et al*. [93] compared the Relevance Network and Bayesian Network models, as well as the Graphical Gaussian Model. The last model is based on the assumption that genes are multivariate Gaussian distributed and the *partial correlation*, calculated on the estimated covariance matrix (through some stabilizing techniques), describes the correlation between two genes. In addition to a *Raf* signaling pathway protein expression data, they run the study using two synthetic data generators (one linear and the other nonlinear) so $N_{G}^{S}$ is known. The evaluation was carried out using 2 criteria: ROC, and comparison of TP given fixed FP value, both of which are based on directed or undirected *edges*, or connections, in the networks *N_I_* and $N_{G}^{S}$. Though the edges carry different meanings in these three different network models, the validation is appropriate in a broader ‘regulation relation’ sense.

Comparing networks of different natures has been attempted by Bansal *et al*. [96]. Three inference procedures were chosen: BANJO (Bayesian network) [97], ARACHE (relevance network) [98], and NIR [99] and MNI [100] (ODE), along with hierarchical clustering and *random* inference which served as references. The choice of these methods was due partially to the availability of their software code. Data were generated from a linear ODE model, and merits of inference were evaluated on the networks using *positive predictive value* (PPV), a ratio of TP and TP+FP, and *sensitivity*, a ratio of TP and TP+FN. Note PPV and *sensitivity* are also referred to as *precision* and *recall* and used by other researchers [101, 102] . It is very interesting to notice the lackluster performances, as demonstrated by being not far from the random method, for all three inference algorithms, particularly on time-series data, using the authors' model setups.

An applaudable endeavor by the DREAM initiative team during the past couple of years allows researchers to validate their networks *N_I_* inferred from the data, which are generated from the *N_G_* the team provides, in a competitive setting [102]. *N_I_* is in the form of pairwise regulation relationships, each tagged with a confidence probability. Much like the work of Werhli *et al*. [93], each participant's inferred network is evaluated against *N_G_* using the area-under-ROC-curve, but with an additional area-under-prediction-versus-recall criterion. It is worth noting that both of these scores are still structure based, and it is expected that the team will have measures that target on the dynamics of inferred networks in the future.

### 4.3. Validation by New Experiments

As a completely different paradigm, the validation can be done experimentally, and the protocol usually runs like this:

1. Inference of network from experiment data;
2. Prediction of certain response using the inferred model;
3. Verification using new experiment.

If the confirmation from new experiment comes back as negative, the model is expected to *learn* from the error and revised. The feedback helps improving the inference procedure, but from a pure validation point of view, this protocol is particularly useful if we are interested in biological network intervention, where the exact correctness of the inferred network structure is of less importance; the ultimate criterion there is whether it can successfully predict the response given a perturbation. In practice the experimental data typically are the responses to selective or systematic perturbation (e.g., stimuli like starvation or drugs), or system behaviors after gene knockout/knock down, and are well suited for network intervention studies.

Such approach has already been taken by researchers. In the study of keratinocyte migration [25], the authors used gene *ptgs2* as migration indicator and built a *recurrent neural network* (RNN) model with nine genes to predict the migration behavior upon hepatocyte growth factor stimulus. They were able to follow it by *in vitro* experiment and the discrepancy with *in silico* predictions helped them build a second RNN model that is more consistent with experimental findings.

## 5. CONCLUSION

We have reviewed the modeling and inference of Gene Regulatory Network (GRN) from time-series data, categorized into those focused on structure, or those on both structure and dynamics, the latter of which is further bifurcated into discrete or continuous space models. The richness of proposed methods calls for comparative performance studies that can be used to establish merit of each inference procedure and appropriateness for a given application. These studies as we see are still lacking despite some modest efforts, due to the fact that ‘ground truth’ network required by various validation schemes is not readily available, and it is not always easy to find a criterion that can effectively evaluate a motley of model types which all have different design goals. On the other hand, the validation can be carried out experimentally where the requirement for ‘ground truth’ network is relaxed. This is especially useful in perturbation-response experiments, and the feedback from new experiments will aid the inferences as well. This survey gives a panoramic view on these topics, with anticipation that the readers will be inspired to improve and/or expand GRN inference and validation tool repository.

## Figures and Tables

**Fig. (1).**
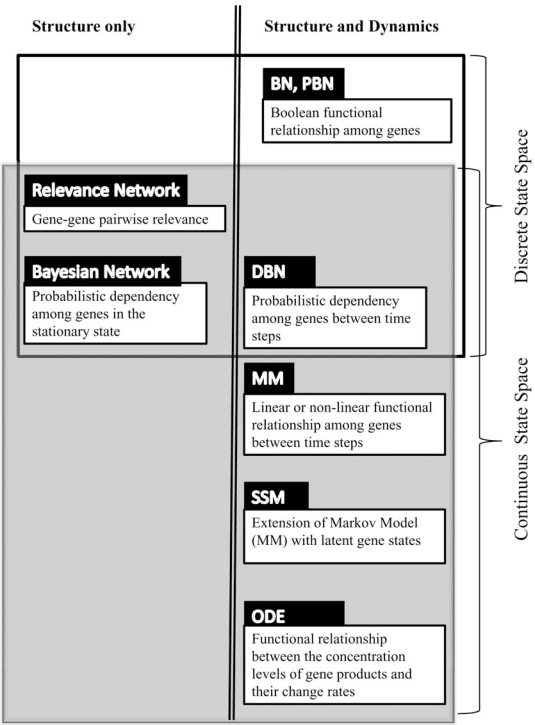
A summary of the models in the paper. Some abbreviations: BN - Boolean Network;  PBN - Probabilistic Boolean Network;  DBN - Dynamic Bayesian Network;  MM - Markov Model;  SSM - State Space Model and ODE - Ordinary Differential Equation.

**Fig. (2).**
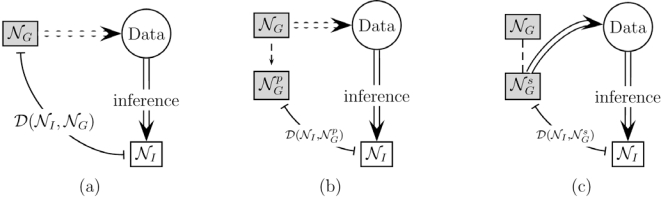
Validation paradigms. $N_{G}^{p}$ and $N_{G}^{s}$ represent the `partial ground truth' and synthetic network, respectively.

**Table 1:** Time Series Data Sets Used to Infer GRNs

Organism	Brief Description	Ref.	Time Points	Year of Publication
B. subtilis	Expression in MMGE environment	[8]	8	1995
Yeast	Diauxie shift; response to medium growth conditions	[9]	7	1997
Yeast	Cell cycle synchronization	[10]	18,24,14*	1998
Yeast	Cell cycle synchronization	[11]	17	1998
Yeast	Yeast Sporulation	[12]	7	1998
House Mouse	Development of the central nervous system of rats	[13]	8	1998
Human	Human Fibroblasts	[14]	13	1999
Yeast	Yeast meiotic expression	[15]	9	2000
E. coli	Tryptophan rich, starving	[16]	8	2000
Drosophila	Life cycle	[17]	74	2002
Human	T-cell activation	[18]	10	2004
Synechocystis	Light intensity experiment	[19]	47,27*	2004
Rat	Circadian rhythmicity of gene expression	[20]	12	2005
B. subtilis	During feed-batch protease production process	[21]	20	2005
E. coli	Perturbation of the SOS system	[22]	6	2006
Human	Endothelial cell apoptosis in blood vessel	[23]	7	2007
Mouse	IL-2-stimulated immune response	[24]	12	2007
Human	Migration of skin keratinocyte	[25]	7	2008

* multiple data sets.
